# Ultrasound‐Assisted Extraction Coupled With Derivatization for Sensitive High‐Performance Liquid Chromatography Determination of Resorcinol in Permanent Hair Dyes

**DOI:** 10.1002/jssc.70441

**Published:** 2026-05-10

**Authors:** Marianna Ntorkou, Kyriaki Letsika, Paraskevas D. Tzanavaras, Constantinos K. Zacharis

**Affiliations:** ^1^ Department of Pharmacy Laboratory of Pharmaceutical Analysis Aristotle University of Thessaloniki Thessaloniki Greece; ^2^ Department of Chemistry Laboratory of Analytical Chemistry Aristotle University of Thessaloniki Thessaloniki Greece

**Keywords:** derivatization, dopamine, hair dye, HPLC, resorcinol, ultrasound‐assisted extraction

## Abstract

Resorcinol is widely used as an oxidative dye intermediate in commercial hair coloring products, yet its accurate quantification remains challenging due to its limited native fluorescence and the strong interferences associated with complex cosmetic matrices. In this study, we report a sensitive and selective high‐performance liquid chromatography–fluorescence method based on dopamine derivatization for the trace‐level determination of resorcinol in hair dye formulations. The analyte was isolated from the sample matrix using ultrasound‐assisted extraction with methanol. Under mild alkaline conditions, dopamine rapidly forms a highly fluorescent adduct with resorcinol, substantially enhancing analytical sensitivity and selectivity. The method was optimized with respect to derivatization parameters, chromatographic separation, and detector response. Validation demonstrated excellent linearity over the range of 1–75 ng/mL (*r* > 0.999), a low limit of detection (= 0.3 ng/mL), and satisfactory recoveries (76.0%–118.0%) across spiked matrices. The robustness of the derivatization procedure was evaluated using Monte‐Carlo simulation experiments. Method greenness was assessed using the multicolor tool, yielding a score of 69.9%. Application to commercial products revealed resorcinol concentrations consistent with reported regulatory limits. The proposed method demonstrates promising potential for routine analytical applications; however, further validation, including inter‐laboratory studies and comparison with established reference methods, is required to support its suitability for regulatory use.

## Graphical abstract

The study presents a sensitive HPLC‐fluorescence method for determining resorcinol in hair dyes using dopamine derivatization and ultrasound‐assisted extraction. The approach enhances selectivity and achieves low detection limits, with validated accuracy and robustness. Application to commercial products confirms compliance with regulations. The method demonstrates strong analytical performance and environmental sustainability, though further inter‐laboratory validation is required for regulatory acceptance.

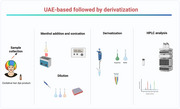

## 1 Introduction

Resorcinol occurs naturally as a monomeric degradation product arising from the reduction, oxidation, and microbial breakdown of humic substances. In addition to its natural formation, it is manufactured globally in substantial quantities and incorporated into a wide range of industrial sectors, including the rubber, adhesive, dye, chemical, and pharmaceutical industries [1, 2]. Within the cosmetics field, resorcinol serves as a key intermediate in oxidative (permanent) hair dye formulations, where it participates in oxidative coupling reactions that generate the final chromophoric structures [3]. Although it plays an important functional role, resorcinol is recognized as an endocrine‐disrupting chemical and is closely regulated due to potential risks associated with dermal penetration, endocrine‐related effects, and sensitization. Accordingly, cosmetic manufacturers are required to indicate their presence on product labels. However, some of them use claims such as “resorcinol‐free” on their packaging to appeal to consumers. As a result, stringent concentration limits such as the maximum permitted level of 1.25% in oxidative hair dye products and 0.5% in pharmaceutical preparations (i.e., shampoo and hair lotions) have been established [4]. Accurate quantification of resorcinol in cosmetic products is therefore essential to ensure product safety and adherence to regulatory standards, including those outlined by the EU Cosmetics Regulation [5].

A variety of techniques have been used to measure resorcinol in various matrices, including liquid or gas chromatography‐tandem mass spectrometry (LC‐MS/MS or GC‐MS/MS) [6, 7], batch spectrofluorimetry [8], high‐performance liquid chromatography‐ultraviolet (HPLC‐UV) [9, 10, 11], micellar electrokinetic chromatography (MEKC) [12, 13], and electrochemistry [14]. Although GC and LC‐MS/MS methods are highly effective, they rely on costly instrumentation, making more affordable and straightforward alternatives highly desirable [15, 16, 17]. Moreover, the high polarity and low volatility 0.03 Pa at 25°C [18]) of resorcinol render GC analysis unsuitable, as it requires prolonged derivatization (approximately 3 h) at relatively high temperatures (i.e., 50°C) [19]. Similar derivatization conditions are also required for LC–MS/MS analysis [7]. Electrochemical techniques are generally sensitive but suffer from low selectivity due to the overlapping oxidation potentials of other phenolic compounds typically present in samples. This limitation necessitates the modification of electrodes with various nanomaterials [20].

To overcome these drawbacks, fluorescence‐based analytical techniques have attracted increasing interest due to their excellent sensitivity, operational simplicity, and compatibility with rapid detection [21, 22, 23]. In particular, the resorcinol‐dopamine reaction provides an effective strategy for achieving highly selective and sensitive resorcinol analysis [8]. This method relies on an oxidative coupling process that generates a strongly fluorescent product known as azamonardine. Under alkaline conditions and in the presence of dissolved oxygen, dopamine is first oxidized to dopaminequinone, which then reacts with resorcinol through a series of nucleophilic substitution and condensation steps. This sequence ultimately yields the azamonardine fluorophore, which exhibits intense blue‐green fluorescence with a typical emission peak in the 450–480 nm range [24].

Effective sample pretreatment is a critical step in chromatographic analysis [25, 26]. Among the most frequently used approaches for analyzing cosmetic products are solvent‐based extraction methods and ultrasound‐assisted procedures [27]. In recent years, however, there has been a growing shift toward techniques that require less sample and reagent consumption while offering simpler equipment and easier handling. Ultrasound‐assisted extraction (UAE) is one such technique that has gained considerable attention and is increasingly becoming routine in analytical laboratories. Ultrasonic energy enhances mass transfer and improves solvent penetration, enabling efficient extraction while markedly reducing both extraction time and solvent usage [28, 29]. In line with this trend, the present study introduces a rapid and environmentally friendly UAE method for the analysis of oxidative hair dye formulations.

Here, we present a novel integrated analytical workflow based on HPLC–spectrofluorimetric method that combines UAE, pre‐column derivatization, and chromatographic separation for the highly sensitive determination of resorcinol in oxidative hair dye formulations. While the dopamine–resorcinol derivatization reaction has been previously reported [30, 31], this study advances the field by embedding this chemistry within a unified UAE‐derivatization‐HPLC‐FLD platform, enabling efficient analyte recovery, enhanced selectivity, and improved applicability to complex cosmetic matrices. Following the extraction of the analyte from the sample matrix, resorcinol is derivatized with dopamine under alkaline conditions to form a fluorescent azamonardine derivative. This product exhibits strong fluorescence, enabling highly sensitive and selective quantification of resorcinol at low ppb levels even within complex cosmetic matrices. The reaction proceeds rapidly at moderate temperatures and does not require specialized catalysts. The stability of the fluorescent product was evaluated and shown to permit accurate measurements for up to 4 h, improving analytical reliability. Derivatization parameters were optimized using a face‐centered central composite design (FC‐CCD), and method validation was conducted in accordance with ICH guidelines. The robustness of the derivatization procedure was investigated using Monte‐Carlo simulation experiments. In addition, the method's environmental impact, operational practicality, analytical performance, and innovative aspects were evaluated using the unified multi‐color assessment (MA) tool. The developed method was successfully applied to hair dye products with minimal pretreatment, demonstrating its suitability as a practical and robust approach for cosmetic and pharmaceutical applications.

## 2 Experimental

### 2.1 Reagents and Solutions

Resorcinol, dopamine, and HPLC‐grade acetonitrile (ACN) (≥98.0%) and methanol (MeOH) were obtained from Merck (Darmstadt, Germany). Ammonium acetate, formic acid (FA) (≥98.0%), sodium carbonate, sodium hydroxide pellets, and all other chemicals were supplied by Sigma‐Aldrich (St. Louis, MO, USA). Ultrapure water was generated using a B30 purification system (Adrona SIA, Riga, Latvia).

Individual stock solutions of resorcinol (1000 µg/mL) and dopamine (1 mM) were prepared in water and kept at 4°C. Working standards were prepared in water from the stock solutions by serial dilution. Sodium carbonate buffer (60 mM) was prepared in water, and the pH was adjusted to 12 using 1 M NaOH solution.

Hair dye products, both resorcinol‐containing and resorcinol‐free, from national and international brands were obtained from local supermarkets and cosmetic suppliers. For method validation, two real matrices confirmed to be free of the target analyte were used. All samples were stored in their original packaging at room temperature until analysis.

### 2.2 Instrumentation and Conditions

A Shimadzu HPLC‐FLD system (Kyoto, Japan) was employed for analysis. The setup comprised a four‐channeled LC‐20AD gradient pump, an RF‐20A fluorescence detector, a SIL‐10AD autosampler, and a CBM‐20A system controller, with system operation managed through LC Solutions software (version 1.25 SP4; Shimadzu). Chromatographic separation was achieved using a Supelcosil C18 analytical column (150 × 4.6 mm, 5 µm) (Supelco, Bellefonte, PA, USA) under isocratic elution conditions. The mobile phases consisted of 0.1% aqueous FA solution (phase A) and MeOH (phase B) 80/20 v/v, delivered at a flow rate of 1 mL/min. The column temperature was 25°C while the injection volume was set to 20 µL. The derivative was monitored spectrofluorimetrically at *λ*_ex_/*λ*_em_ = 420/460 nm using high sensitivity mode. An Elmasonic Easy 30H (Elma Schmidbauer GmbH, Singen, Germany) operated at 37 kHz, and 80 W was utilized through this study.

### 2.3 Sample Collection and UAE Procedure

For each analysis, the first portion of hair coloring cream (approximately 5 cm from the tube) was discarded to avoid potential oxidation caused by exposure to air. A quantity of 500 mg of the sample was accurately weighed and transferred to a 50 mL volumetric flask. The sample was spiked with 1000 µL of the analyte solution to achieve the target concentration, and the flask was filled to volume with MeOH. The mixture was vortexed for 30 s, followed by sonication in an ultrasonic bath (80 W, 37 kHz) for 5 min at 25°C. Before the derivatization step, the extract was diluted either 100‐ or 500‐fold with MeOH, depending on the required concentration range.

### 2.4 Derivatization Protocol

An aliquot of 200 µL of diluted sample was mixed with 300 µL of an aqueous dopamine solution (1 mM), and 500 µL of sodium carbonate buffer (60 mM, pH 12.0) in an Eppendorf tube. The resulting mixture was vortex‐mixed for 5 s, allowed to react for 1 min at ambient temperature, and subsequently analysed using HPLC‐FLD.

## 3 Results and Discussion

### 3.1 Resorcinol‐Dopamine Reaction Mechanism

**FIGURE 1:**
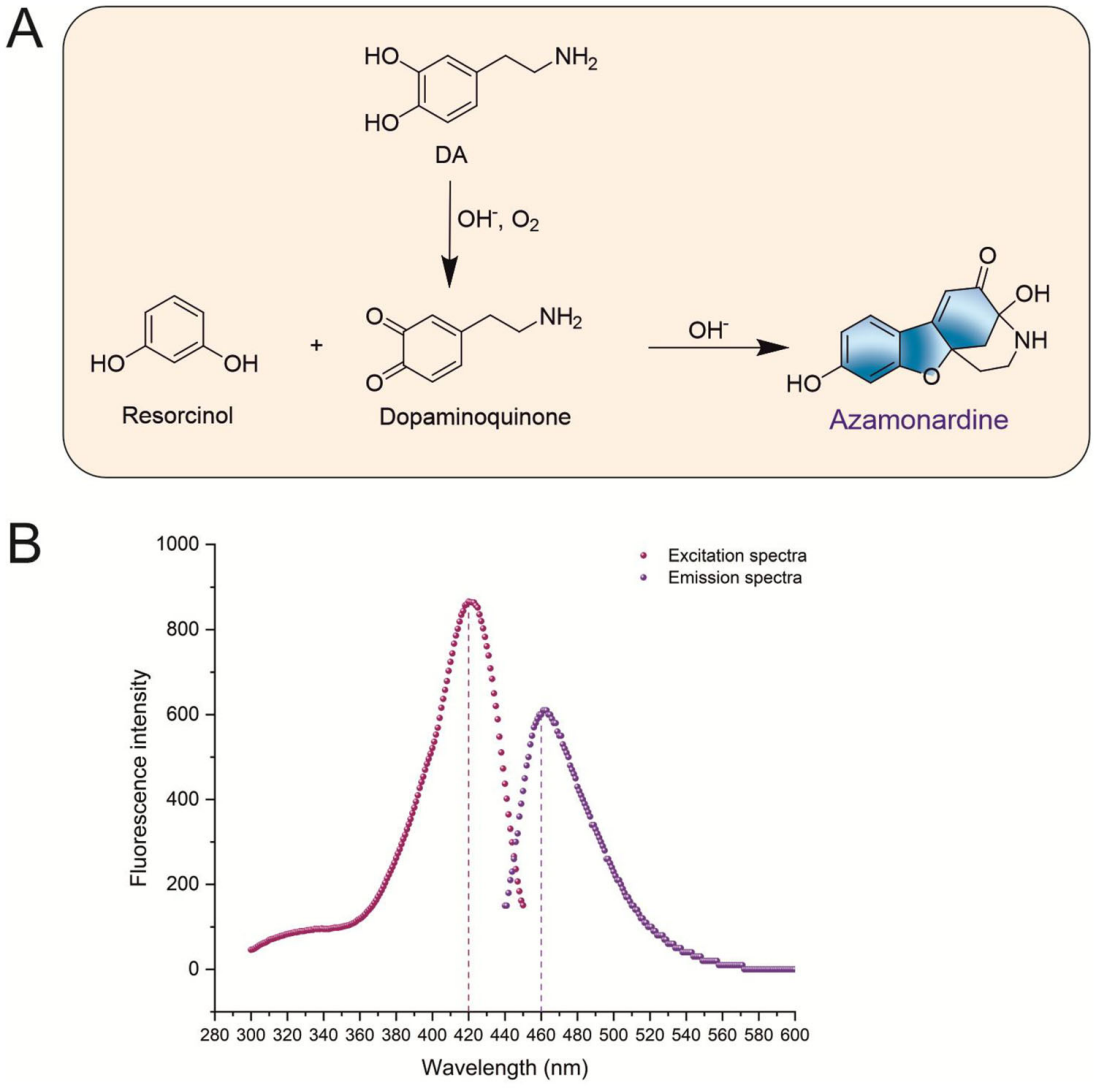
**FIGURE 1** (A) Reaction scheme of resorcinol with dopamine in an alkaline medium. (B) Excitation and emission spectra of azamonardine derivative.

The reaction of resorcinol with dopamine to form fluorescent monardine or azamonardine derivatives is well documented [32]. In this work, we employed the same reaction pathway to generate a stable fluorescent compound, azamonardine, for the determination of resorcinol (Figure 1A). From a mechanistic standpoint, dopamine is readily oxidized under alkaline conditions to produce dopaminoquinone. Although dopamine slowly self‐polymerizes at pH 11.5 and forms a pale‐yellow product after 30 min, the resulting polymer exhibits little to no fluorescence [33]. Resorcinol, however, does not oxidize to a quinone in this environment. Instead, it undergoes deprotonation to generate two monoanionic resonance structures, which stabilize a reactive oxygen anion [34]. This anionic species then performs a nucleophilic attack on the electrophilic quinone ring of dopaminoquinone, forming a tricyclic intermediate containing a five‐membered oxygen heterocycle. A subsequent nucleophilic addition by either the hydroxyl or amino group on dopaminoquinone to the intermediate's carbonyl group ultimately yields the highly fluorescent azamonardine molecule [32].

Following the reaction between resorcinol and dopamine, the solution turned pale yellow and displayed intense blue fluorescence excited at 420 nm. The fluorescence spectra are depicted in Figure 1B. These findings are aligned with the existing data [8].

### 3.2 HPLC‐FLD Method Development

A key advantage of this reaction is that blank mixtures exhibit no background fluorescence, meaning that separation conditions do not require extensive optimization beyond enhancing peak shape and chromatographic efficiency of the derivative. Based on this, various mobile phase compositions were tested, including phosphate buffer at pH 7, 0.1% aqueous FA combined with either MeOH or ACN. The experimental findings showed that acidic mobile phases yielded better peak shapes than those at neutral pH, likely because protonation of the azamonardine derivative's secondary amino group reduces electrostatic interactions with ionized silanol groups on the stationary phase. As a result, 0.1% FA was chosen as the aqueous component of the mobile phase. Operating under acidic conditions also helps neutralize the alkaline sample, thereby prolonging the stationary phase's lifespan. MeOH and ACN produced similar fluorescence intensities for the azamonardine derivative. Therefore, MeOH was selected for its lower cost and more favorable environmental footprint. Consequently, a mobile phase of 0.1% v/v aqueous FA and MeOH (80/20, v/v) was chosen for further analysis, offering an appropriate retention time and satisfactory peak shape.

### 3.3 Optimization of UAE Conditions

To optimize the UAE procedure, the effects of extraction solvent (MeOH vs. ACN) and sonication time (2, 5, and 10 min) were evaluated. Both solvents yielded nearly quantitative extraction after 10 min of sonication. Given their similar performance, MeOH was chosen based on its lower cost and reduced environmental impact. A 36% increase in extraction recovery was observed when extending sonication from 2 to 5 min, with no substantial improvement beyond that point. Consequently, a 5 min sonication time span was selected for subsequent experiments.

### 3.4 Optimization of the Derivatization Conditions

**FIGURE 2:**
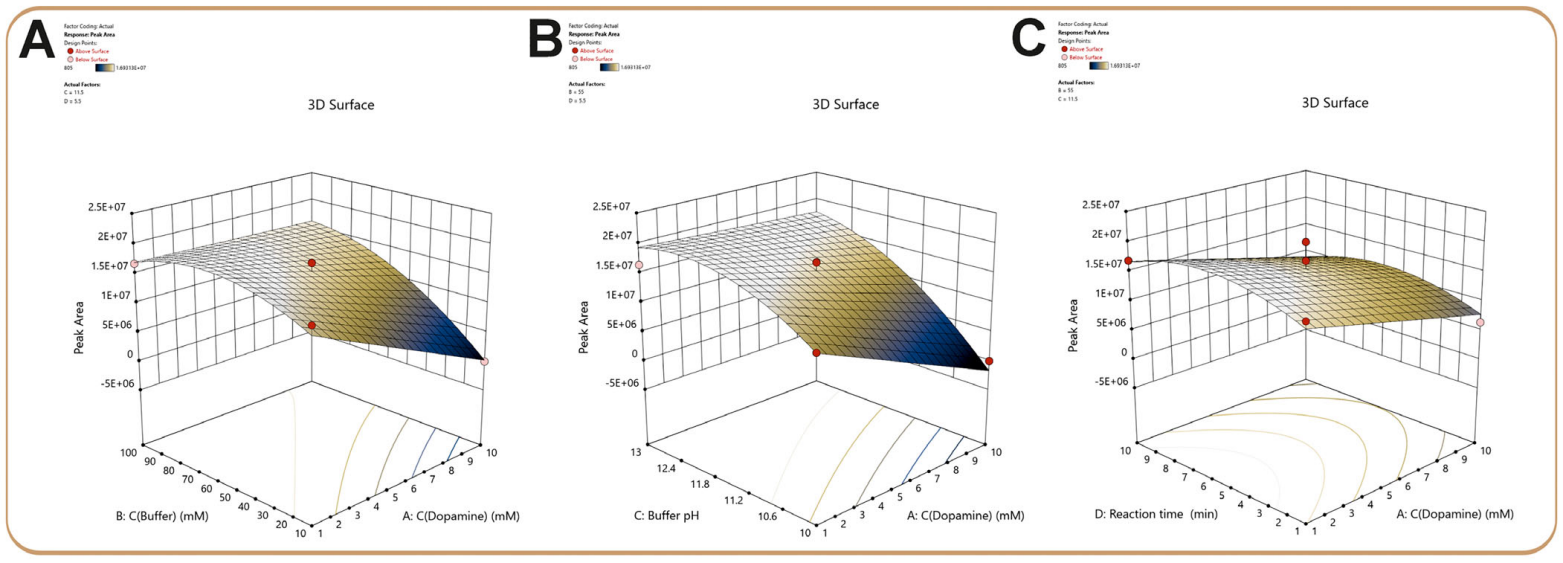
3D plots of the effect of (A) buffer and dopamine concentrations, (B) buffer pH and dopamine concentration, and (C) reaction time and dopamine concentration on the resorcinol derivative peak area.

A response surface methodology using FC‐CCD was applied to evaluate and optimize the reaction yield by examining the effects of dopamine concentration, buffer concentration, buffer pH, and reaction time. The design consisted of 29 randomized experiments (16 factorial points, eight axial points, and five center points). The dataset is shown in Table S1, allowing robust assessment of both linear and quadratic factors. Data analysis was performed using Design Expert 13 (Stat‐Ease Inc., Minneapolis, MN, USA). ANOVA results (Table S2) indicated that the dopamine and buffer concentration and the buffer pH had the greatest influence on the derivative's peak area, underscoring their critical roles in the derivatization process. The effect of the reaction time was minimal in the studied range of 1–10 min. The model exhibited strong predictive capability, with *R* ^2^ and adjusted *R* ^2^ values of 0.8922 and 0.8411, respectively. Non‐significant lack‐of‐fit result (*p* > 0.05) further supported the model's validity. 3D response surface plots (Figure 2) visualized the influence of each factor on azamonardine peak area. The adequate precision value was 13.6, which is higher than 4, indicating the significance of the model. Diagnostic plots such as the normal probability plot of residuals and the plot of residuals against the predicted values are portrayed in Figure S1. The data were randomly scattered around the line, indicating that the model was properly fitting the data. The variability of the actual model residuals was compared with that of replicate settings, and the lack‐of‐fit parameter (*p* = 0.0611) was found to be non‐significant (*p* > 0.05). The predicted coded model was expressed from the following mathematical formula:

$$\begin{array}{ccc} Peak\;Area & = & \left(1.5\pm 0.2\right)\times 10^{7}-\left(3.9\pm 1.7\right)\\ & & \times \;10^{6}A+\left(4.5\pm 1.7\right)\times 10^{6}B+\left(6.6\pm 1.7\right)\times 10^{6}C\\ & & +\;\left(5.4\pm 3.2\right)\times 10^{5}D+\left(3.5\pm 2.9\right)\times 10^{6}AB\\ & & +\;\left(3.1\pm 2.9\right)\times 10^{6}\times AC-\left(3.4\pm 2.2\right)\times 10^{6}B^{2}\\ & & -\;\left(3.6\pm 2.3\right)\times 10^{6}C^{2}-\left(3.2\pm 2.3\right)\times 10^{6}D^{2} \end{array}$$

where A, B, C, and D are the concentrations of dopamine, buffer concentration, buffer pH, and reaction time, respectively.

Optimal conditions were established using Derringer's desirability function, aiming to maximize the derivative peak area while minimizing both the reaction time and the dopamine concentration. The latter was minimized in accordance with Green Analytical Chemistry principles, which emphasize reducing reagent consumption whenever possible [35]. This optimization produced an overall desirability score of 0.992 and identified an optimal dopamine concentration of 1 mM, a reaction time of 1 min, with a buffer concentration of 60 mM and pH of 12 (rounded) (Figures S2 and S3). The desirability reflects a compromise between maximizing peak area and minimizing derivatization time and dopamine concentration, which naturally oppose each other. Model verification with six replicates at 50 ng/mL confirmed its accuracy, with predicted‐to‐observed peak areas ranging from 94% to 103% at the 95% confidence level.

### 3.5 Analytical Method Validation

The developed HPLC‐FLD method was validated using the ICH guidelines [36] by investigating the parameters, including specificity, linearity, precision, accuracy, limit of detection (LOD), limit of quantitation (LOQ), and robustness.

Two hair dye matrices advertised as resorcinol‐free were used to assess the selectivity of the proposed method. Both samples exhibited several peaks within the 1.5–2.5 min window, corresponding to other formulation excipients. However, no signal appeared at the retention time of the resorcinol derivative (ca 6.2 min), confirming the absence of interfering components. To further evaluate potential matrix effects, these resorcinol‐free samples were fortified with 10 ng/mL resorcinol and subsequently analyzed (Figure S4). The combination of selective derivatization and chromatographic separation significantly reduces the likelihood that matrix interferences contribute to the detected signal. Nevertheless, while standard addition supports accuracy, it does not definitively confirm analyte identity and is therefore considered complementary.

Carryover effects were evaluated by sequentially analyzing a resorcinol‐free sample following the analysis of a blank sample spiked at the highest calibration level. The resulting peak area was lower than 15% of that observed at the lower LOQ level, confirming that the autosampler was efficiently washed between analyses.

Linearity was assessed using resorcinol standard solutions spanning concentrations from 1 to 75 ng/mL. Calibration parameters, including slope, intercept, and the correlation coefficient (*r*), were determined by plotting the derivative's peak area against resorcinol concentration using least‐squares linear regression. The resulting calibration data are presented in Table 1. Excellent linearity was achieved for the analyte, with an *r* value exceeding 0.9994.

**TABLE 1:** Analytical figures of merit of the developed method for the determination of resorcinol.

Linear range	Calibration curve [PA = (*A*±SD_A_)C + (*B*±SD_B_), (*r*)]^a^	LOD^b^ (ng/mL)	LOQ^c^ (ng/mL)
1–75	PA = (19521 ± 155)C + (27431 ± 5978), (0.9994)	0.3	1

Nominal concentration (ng/mL)	Intra‐day (*n* = 3) Recovery (%RSD)	Inter‐day (*n* = 3) Recovery (%RSD)	
10	117.6 (2.2)	97.6 (18)	
25	100.7 (3.9)	85.8 (15.3)	
50	110.7 (5.1)	105.3 (4.4)	

^a^ PA: Peal area, A: slope, SD_A_: standard deviation of slope, B: intercept, SD_B_: standard deviation of intercept, *r*: correlation coefficient.

^b^ LOD: Based on S/N = 3.

^c^ LOQ: Based on S/N = 10.

The intra‐ and inter‐day trueness and precision of the analytes were evaluated using blank hair dye samples spiked at three concentration levels at 10, 25, and 50 ng/mL corresponding to 500, 1250, and 2500 µg/g, respectively. Intra‐day precision was determined by three consecutive analyses of each concentration within a single day. Inter‐day precision was calculated from the average of three intra‐day measurements collected on three non‐consecutive days.

Trueness was expressed as the recovery, defined as the closeness between the measured mean concentration and the spiked true value, whereas precision was reported as the relative standard deviation (RSD) of the measured concentrations. The results, summarized in Table 1, showed recoveries (85.6%–117.6%) within the commonly accepted range of 80%–120%, while precision (≤ 18%) met the typical criterion of ≤ 20% for complex matrices. These acceptance criteria were selected in accordance with established validation guidelines and literature practices for trace‐level analysis in cosmetic samples [37]. The LOD and LOQ were determined using a signal‐to‐noise (*S*/*N*) ratio of 3 and 10, and were found to be 0.3 and 1 ng/mL, corresponding to 16 and 50 µg/g, respectively.

The robustness of the derivatization procedure was assessed through Monte Carlo simulations and process capability analysis. A total of 100 000 simulated iterations were generated, and the resulting data were used to calculate *Cpk* values. An acceptance range of ±5% relative to the predicted peak area from the optimization stage was defined for the resorcinol signal. Simulations were performed using mean values of 1 mM dopamine, 61.7 mM buffer concentration, pH 12.2, and a reaction time of 1 min, with corresponding standard deviations of 0.1, 10, 0.2, and 0.1, respectively. The capability analysis yielded a *Cpk* of 1.34, indicating that only 0.02% of outcomes would fall outside the specification limits, which meet the commonly accepted threshold (≥1.33). The histogram representing the capability analysis of the azamonardine peak area is shown in Figure S5.

System suitability is an essential parameter that ensures the chromatographic system provides adequate resolution and reproducibility for analysis and confirms whether the developed method is valid. The acceptance criteria were defined such that the number of theoretical plates should be at least 5000, while the tailing factor should fall within the range of 0.8–1.8.

### 3.6 Derivative Stability

The stability of azamonardine was assessed over a 4 h period, a crucial parameter for ensuring reliable analysis when derivatized samples are held in the autosampler at room temperature during batch runs. The experimental data revealed that the derivative demonstrated excellent stability, with an RSD of 1.6% and no meaningful fluctuations in peak area or signs of degradation. These results confirm that derivatized samples remain suitable for accurate quantification within this time window.

### 3.7 Application to Commercial Hair Dye Samples

The applicability of the proposed HPLC‐FLD method was tested by analyzing commercial hair dye products intended for women and men at various colors (dark, blonde, brown shades, etc.). The processing of the samples is described in detail in Section 2.3. Each sample set was analyzed in triplicate, and the resulting data are summarized in Table 2. Resorcinol was detected in all products at concentrations ranging from 194 to 655 µg/g. All products analyzed EU Regulation restrictions fulfilled.

**TABLE 2:** Relative recoveries of resorcinol performed in five branded oxidative hair dye samples.

Sample	Added Concentration (µg/g)	Found concentration (µg/g)	Relative Recoveries (%) (RSD%, *n* = 3)
A	—	655	—
	500	1245	118.0 (2.2)
	1250	1915	100.8 (3.9)
	2500	3425	110.8 (4.4)
B	—	240	—
	500	665	85.0 (3.8)
	1250	1255	81.2 (4.9)
	2500	2805	102.6 (3.4)
C^a^	—	194	—
	500	649	91.0 (4.6)
	1250	1184	79.2 (5.0)
	2500	2429	89.4 (4.4)
D	—	75	—
	500	475	80.0 (4.6)
	1250	1025	76.0 (5.3)
	2500	2220	85.8 (6.3)
E	—	315	—
	500	765	90.0 (6.9)
	1250	1395	86.4 (5.9)
	2500	2765	98.0 (6.8)

^a^ Diluted 100‐fold.

**FIGURE 3:**
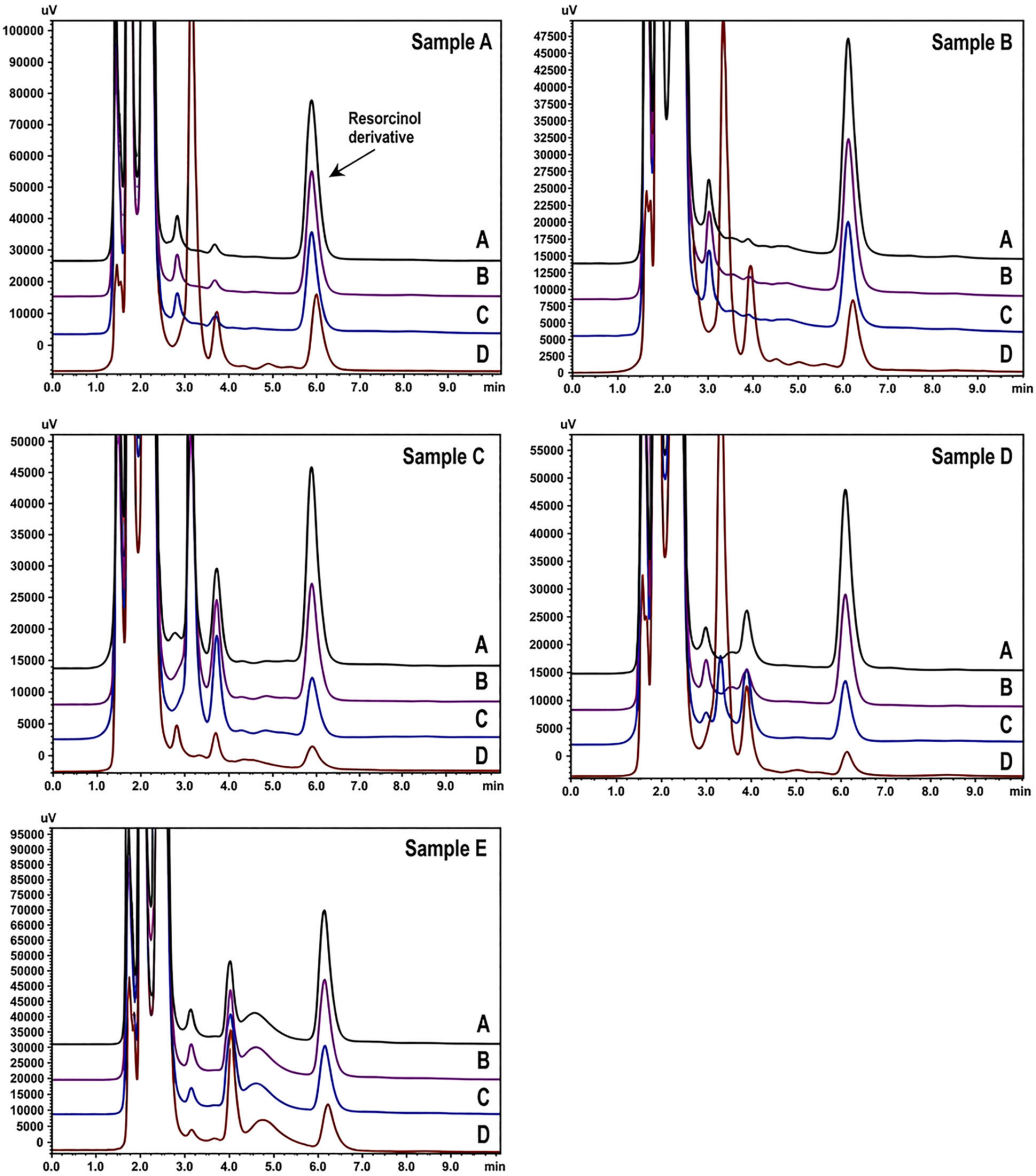
High‐performance liquid chromatography–fluorescence detection (HPLC‐FLD) chromatograms of the analysis of branded hair dye samples A–E. Sample spiked at (A) 2500 µg/g, (B) 1250 µg/g, and (C) 500 µg/g of the analyte.

Spiked recovery tests were also performed by fortifying the real samples with concentration levels of 500, 1250, and 2500 µg/g of the resorcinol standard solution. All samples exhibited recovery values in the range of 76.0%–118.0%. The observed variability can be attributed to the multi‐step nature of the procedure, including extraction and derivatization, which may influence analyte recovery, particularly at trace levels. Representative HPLC chromatograms from the analysis of samples are illustrated in Figure 3. The experimental findings demonstrated that the method offers high accuracy and strong reproducibility, confirming its suitability for the quantitative determination of resorcinol in real samples.

### 3.8 Comparison With Other Published Approaches

The performance of the proposed method was compared with representative previously reported techniques for determining the analyte in cosmetics and biological samples (Table 3). Methods employing GC‐MS/MS and LC‐MS/MS generally demonstrated high sensitivity, with LOD down to 0.061 µg/g and method precision below 9% [6, 7]. Spectrofluorimetric and HPLC‐UV methods offered simple workflows‐often relying only on dilution or traditional liquid–liquid extraction (LLE) but showed higher LODs (70–950 ng/mL), despite maintaining good precision (<3.3% RSD in most cases) [8, 10, 11]. MEKC achieved moderate sensitivity, with LODs ranging from 17.8 to 310 ng/mL and consistently low RSD values [12, 13]. The proposed approach, which integrates UAE with dopamine derivatization and HPLC‐FLD, provided one of the most sensitive non‐MS detection methods reported to date (LOD = 0.3 ng/mL), albeit with slightly higher variability (<18% RSD). Overall, methods using mass spectrometry offered superior sensitivity, while spectroscopic and chromatographic UV‐based approaches balanced simplicity and precision. The new UAE–based HPLC‐FLD method stands out as a highly sensitive alternative suitable for complex cosmetic matrices.

**TABLE 3:** Comparison of the proposed method with other published approaches for the determination of resorcinol in cosmetics and biological samples.

Sample	Method principle^a^	Analytical technique	LOD	Precision (%RSD)	%R	Ref.
Oxidative hair dyes	Comparison of VE, UAE, and MSPD	GC‐MS/MS	0.061 µg/g	<7.4	56.3–101	[6]
Human urine	Sample hydrolysis followed by SLE with ethyl acetate and derivatization with Dansyl‐Cl	LC‐MS/MS	NM^b^	<8.65	95.6–105	[7]
Hair dye, wheat flour	Derivatization with dopamine	Batch spectrofluorimetry	22 ng/mL	<3.34	96.74–100.38	[8]
Cosmetic products	Dilution with water and MeOH	HPLC‐UV	70 ng/mL	<3.3	98.4–101.6	[9]
Hair dye products	LLE with ether, evaporation, and reconstitution	HPLC‐UV	950 ng/mL	<2.2	97–99	[10]
Hair tonic	LLE with chloroform, evaporation, and reconstitution	HPLC‐UV	630 ng/mL	<0.89	80.76–121.29	[11]
Moisturizer, lotion, cream	Dilution with ethanol	MEKC	310 ng/mL	<2.7	99.0	[12]
Mask, golden essence, lotion, facial washing milk	Dilution with ethanol	MEKC	17.8 ng/mL	<4.94	91.4–107.4	[13]
**Oxidative hair dye**	**UAE followed by derivatization with dopamine**	**HPLC‐FLD**	**0.3 ng/mL (16 µg/g)**	**<18**	**76.0–118.0**	**This study**

^a^ VE: Vortex extraction; UAE: Ultrasound‐assisted extraction; MSPD: matrix solid‐phase dispersion; SLE: supported‐liquid extraction; LLE: liquid‐liquid extraction.

^b^ NM: not mentioned.

### 3.9 Greenness Assessment Using the Unified MA Tool

The environmental performance of the developed method was evaluated using the unified MA tool [38], an integrated online platform that consolidates four established assessment frameworks into a single system. This platform merges the Green Evaluation Metric for Analytical Methods (GEMAM), the Blueness Assessment Graphical Index (BAGI), the Redness Analytical Performance Index (RAPI), and the Violet Innovation Grade Index (VIGI), using a comprehensive 51‐item questionnaire.

**FIGURE 4:**
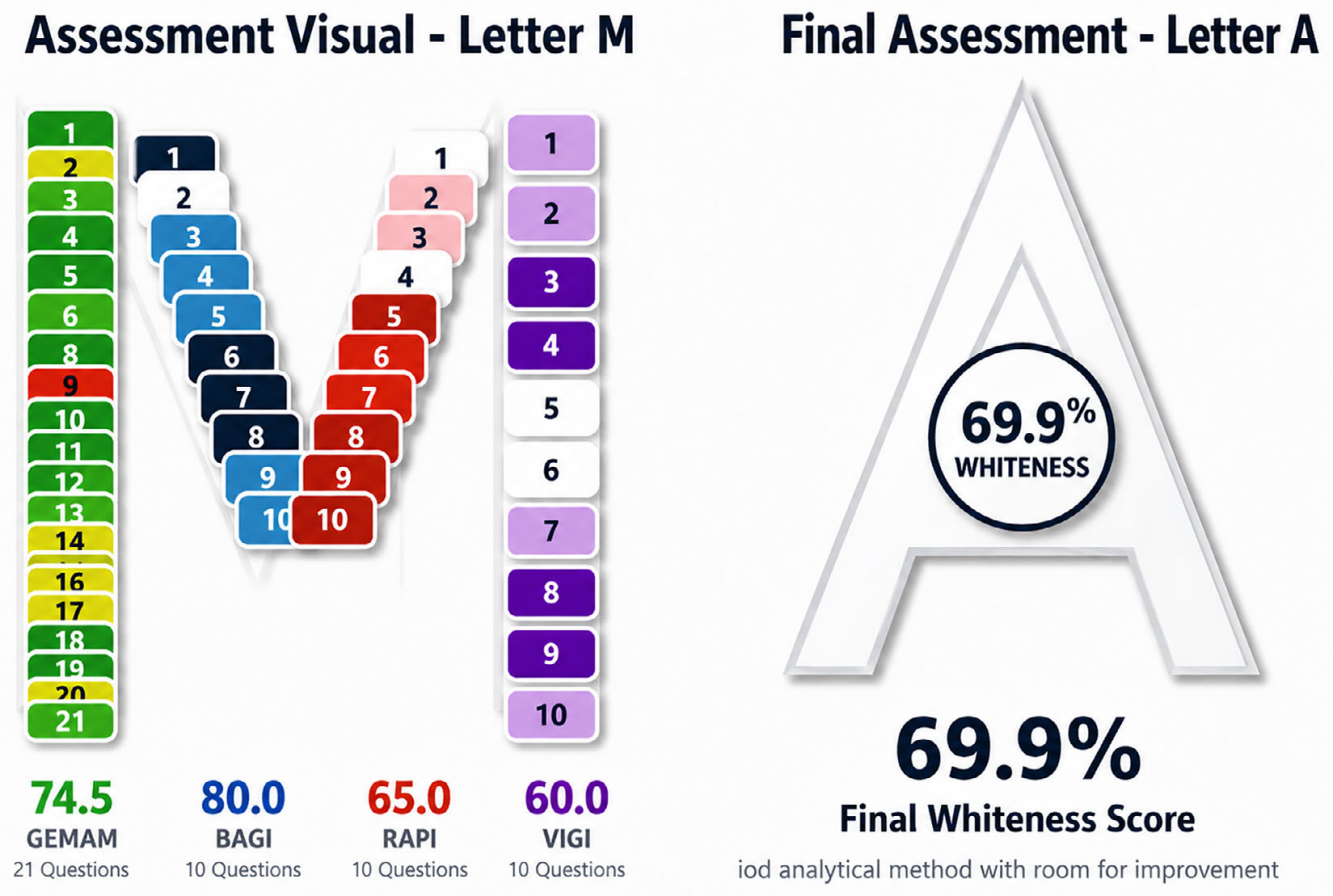
Multi‐color assessment (MA) whiteness assessment score of the proposed high‐performance liquid chromatography–fluorescence detection (HPLC‐FLD) method for the determination of resorcinol.

The method demonstrated strong environmental sustainability (GEMAM: 74.5%), largely due to the implementation of advanced sample preparation techniques (Figure 4). It also showed excellent practical applicability (BAGI: 80.0%), notable innovative character (VIGI: 60.0%), and good compliance with White Analytical Chemistry (WAC) principles. Analytical performance was found to be satisfactory (RAPI: 65.0%). Overall, the method achieved an MA Whiteness Score of 69.9%, indicating favorable sustainability attributes and highlighting key strengths in innovation and practical use.

A comparison with reported methods (Table 3) indicates that conventional approaches (e.g., HPLC‐UV and GC‐MS/MS) often require solvent‐intensive sample preparation such as liquid–liquid extraction with organic solvents and evaporation steps, increasing environmental impact and analysis time. In contrast, the proposed method employs UAE, reducing solvent consumption and simplifying the workflow, contributing to improved greenness.

## 4 Conclusions

In conclusion, this study introduces a rigorously optimized and analytically robust HPLC–spectrofluorimetric method for trace‐level determination of resorcinol in oxidative hair dye formulations. The coupling of ultrasound‐assisted extraction with rapid dopamine‐based derivatization enables the formation of a stable, highly fluorescent adduct, effectively addressing the challenges posed by complex cosmetic matrices. Validation in accordance with ICH guidelines and optimization via FC‐CCD confirm the method's precision, sensitivity, and reliability, while the MA highlights its favorable environmental and operational profile. Successful application to commercial products demonstrates the method's practicality and potential for routine analysis in cosmetic and pharmaceutical settings; however, further validation, including inter‐laboratory studies and comparison with established reference methods, is required to fully support its broader applicability.

## Author Contributions

**Marianna Ntorkou**: data curation, investigation, formal analysis, validation, and writing – original draft. **Kyriaki Letsika**: formal analysis and validation. **Constantinos K. Zacharis**: conceptualization, methodology, investigation, supervision, writing – original draft, writing – review and editing, visualization, resources, and software. **Paraskevas D. Tzanavaras**: writing – review and editing.

## Conflicts of Interest

The authors declare no conflicts of interest.

## Supporting information




**Supporting File**: jssc70441‐sup‐0001‐SuppMat.docx.

## Data Availability

The data that support the findings of this study are available from the corresponding author upon reasonable request.
