# Supplementary material for: Ultrasound‐Assisted Extraction Coupled With Derivatization for Sensitive High‐Performance Liquid Chromatography Determination of Resorcinol in Permanent Hair Dyes
Source: J Sep Sci. 2026 May 10;49:e70441. doi: 10.1002/jssc.70441 (PMC13158550; doi:10.1002/jssc.70441)
Supplement: Supplementary file 1 — Supporting File: jssc70441‐sup‐0001‐SuppMat.docx. [file JSSC-49-e70441-s001.docx]

**Supplementary material**

**Ultrasound-Assisted Extraction Coupled with Derivatization for Sensitive HPLC Determination of Resorcinol in Permanent Hair Dyes**

Marianna Ntorkou^1^, Kyriaki Letsika^1^, Paraskevas D. Tzanavaras^2^, Constantinos K. Zacharis^1 *^

*^1^ Laboratory of Pharmaceutical Analysis, Department of Pharmacy, Aristotle University of Thessaloniki, GR-54124, Greece*

*^2^ Laboratory of Analytical Chemistry, Department of Chemistry, Aristotle University of Thessaloniki, GR-54124, Greece*

*Corresponding author

Constantinos K. Zacharis

Associate Professor

Laboratory of Pharmaceutical Analysis, School of Pharmacy,

Aristotle University of Thessaloniki (AUTh),

GR-54124, Greece

Tel: +30 2310997663

E-mail: [czacharis@pharm.auth.gr](mailto:czacharis@pharm.auth.gr)

**Table S1**. FC-CCD dataset for the optimization of derivatization conditions.

| **Run No** | **C(Dopamine) (mM)**  **(Factor 1)** | **Buffer concentration (mM)**  **(Factor 2)** | **Buffer pH**  **(Factor 3)** | **Reaction time (min)**  **(Factor 4)** | **Peak Area** |
| --- | --- | --- | --- | --- | --- |
| 1 | 10 | 10 | 11.5 | 5.5 | 1411 |
| 2 | 5.5 | 55 | 11.5 | 5.5 | 16836016 |
| 3 | 10 | 55 | 11.5 | 10 | 11809797 |
| 4 | 5.5 | 55 | 13 | 10 | 16672351 |
| 5 | 5.5 | 100 | 11.5 | 10 | 16681784 |
| 6 | 5.5 | 55 | 11.5 | 5.5 | 15370706 |
| 7 | 5.5 | 55 | 11.5 | 5.5 | 14039699 |
| 8 | 5.5 | 100 | 10 | 5.5 | 5290361 |
| 9 | 5.5 | 10 | 11.5 | 1 | 1025 |
| 10 | 1 | 55 | 13 | 5.5 | 16438803 |
| 11 | 5.5 | 55 | 10 | 10 | 530566 |
| 12 | 1 | 55 | 10 | 5.5 | 12245447 |
| 13 | 5.5 | 10 | 10 | 5.5 | 805 |
| 14 | 5.5 | 100 | 11.5 | 1 | 16276567 |
| 15 | 1 | 10 | 11.5 | 5.5 | 16396166 |
| 16 | 5.5 | 55 | 10 | 1 | 45897 |
| 17 | 10 | 55 | 13 | 5.5 | 16488225 |
| 18 | 5.5 | 10 | 11.5 | 10 | 11648 |
| 19 | 10 | 100 | 11.5 | 5.5 | 14162390 |
| 20 | 10 | 55 | 10 | 5.5 | 3413 |
| 21 | 5.5 | 55 | 11.5 | 5.5 | 14407839 |
| 22 | 1 | 100 | 11.5 | 5.5 | 16727630 |
| 23 | 5.5 | 100 | 13 | 5.5 | 16289546 |
| 24 | 10 | 55 | 11.5 | 1 | 6452882 |
| 25 | 1 | 55 | 11.5 | 10 | 16905446 |
| 26 | 1 | 55 | 11.5 | 1 | 16695864 |
| 27 | 5.5 | 55 | 11.5 | 5.5 | 16931294 |
| 28 | 5.5 | 55 | 13 | 1 | 16678891 |
| 29 | 5.5 | 10 | 13 | 5.5 | 15274077 |

**Table S2**. ANOVA table for the peak area of azamonardine derivative.

| **Source** | **Sum of Squares** | **df** | **Mean Square** | **F-value** | **p-value** |  |
| --- | --- | --- | --- | --- | --- | --- |
| **Model** | 1.220E+15 | 9 | 1.355E+14 | 17.47 | < 0.0001 | significant |
| A-C(Dopamine) | 1.801E+14 | 1 | 1.801E+14 | 23.22 | 0.0001 |  |
| B-C(Buffer) | 2.407E+14 | 1 | 2.407E+14 | 31.02 | < 0.0001 |  |
| C-Buffer pH | 5.297E+14 | 1 | 5.297E+14 | 68.27 | < 0.0001 |  |
| D-Reaction time | 3.478E+12 | 1 | 3.478E+12 | 0.4483 | 0.5112 |  |
| AB | 4.781E+13 | 1 | 4.781E+13 | 6.16 | 0.0226 |  |
| AC | 3.777E+13 | 1 | 3.777E+13 | 4.87 | 0.0399 |  |
| B² | 7.988E+13 | 1 | 7.988E+13 | 10.30 | 0.0046 |  |
| C² | 8.671E+13 | 1 | 8.671E+13 | 11.18 | 0.0034 |  |
| D² | 7.060E+13 | 1 | 7.060E+13 | 9.10 | 0.0071 |  |
| **Residual** | 1.474E+14 | 19 | 7.759E+12 |  |  |  |
| Lack of Fit | 1.402E+14 | 15 | 9.349E+12 | 5.21 | 0.0611 | not significant |
| Pure Error | 7.174E+12 | 4 | 1.794E+12 |  |  |  |
| **Cor Total** | 1.367E+15 | 28 |  |  |  |  |

^1^degree of freedom


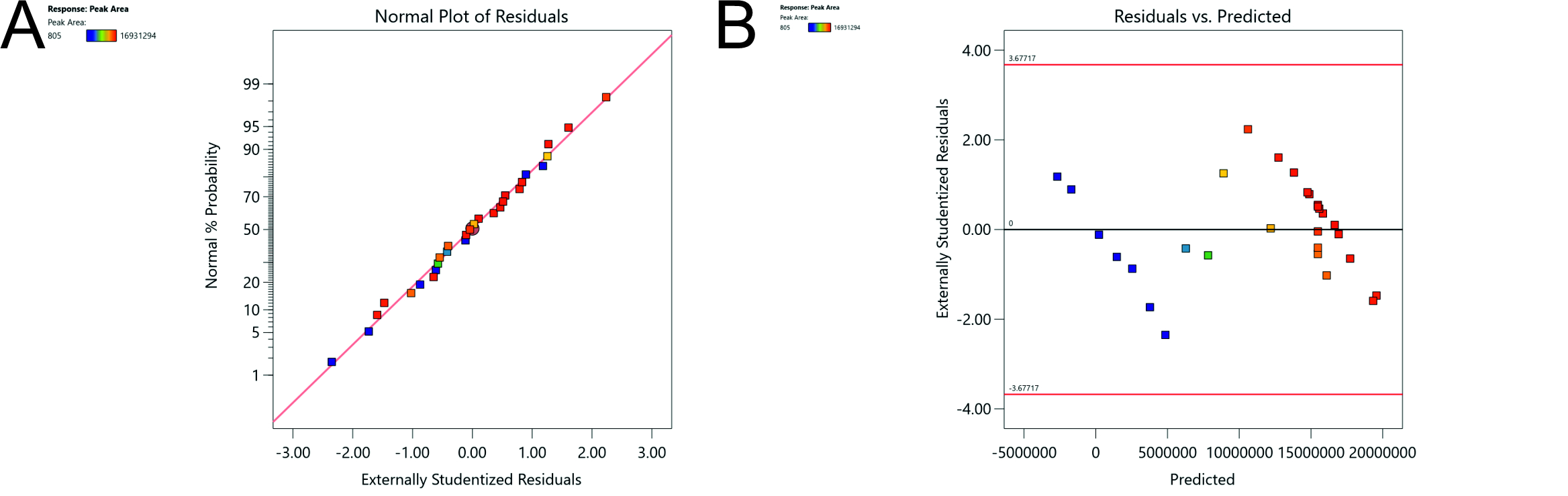


**Figure S1.** Normal probability and the residuals vs predicted plots for the azamonardine peak area.


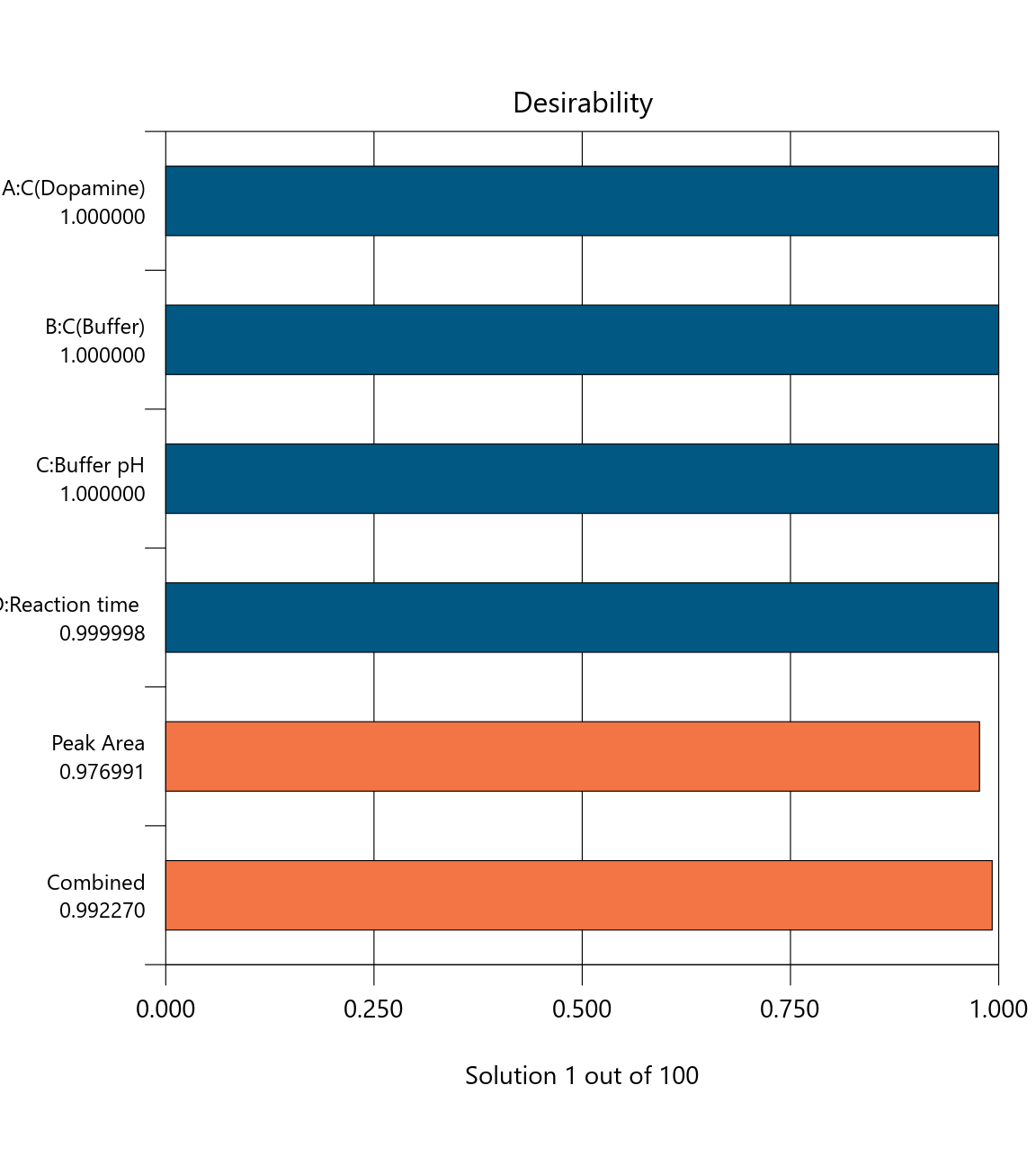


**Figure S2**. Desirability data of each parameter studied.


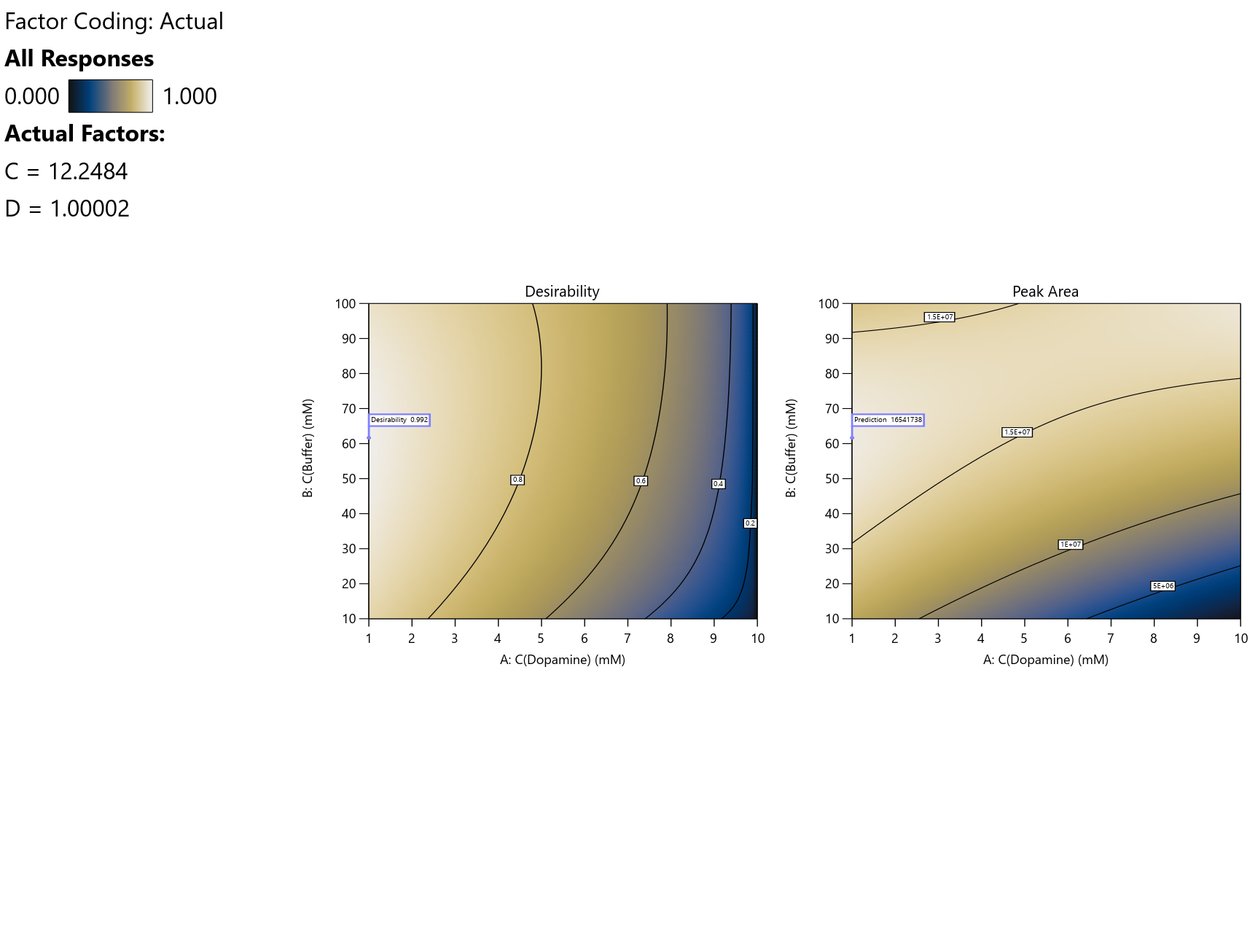


**Figure S3.** Desirability plots.


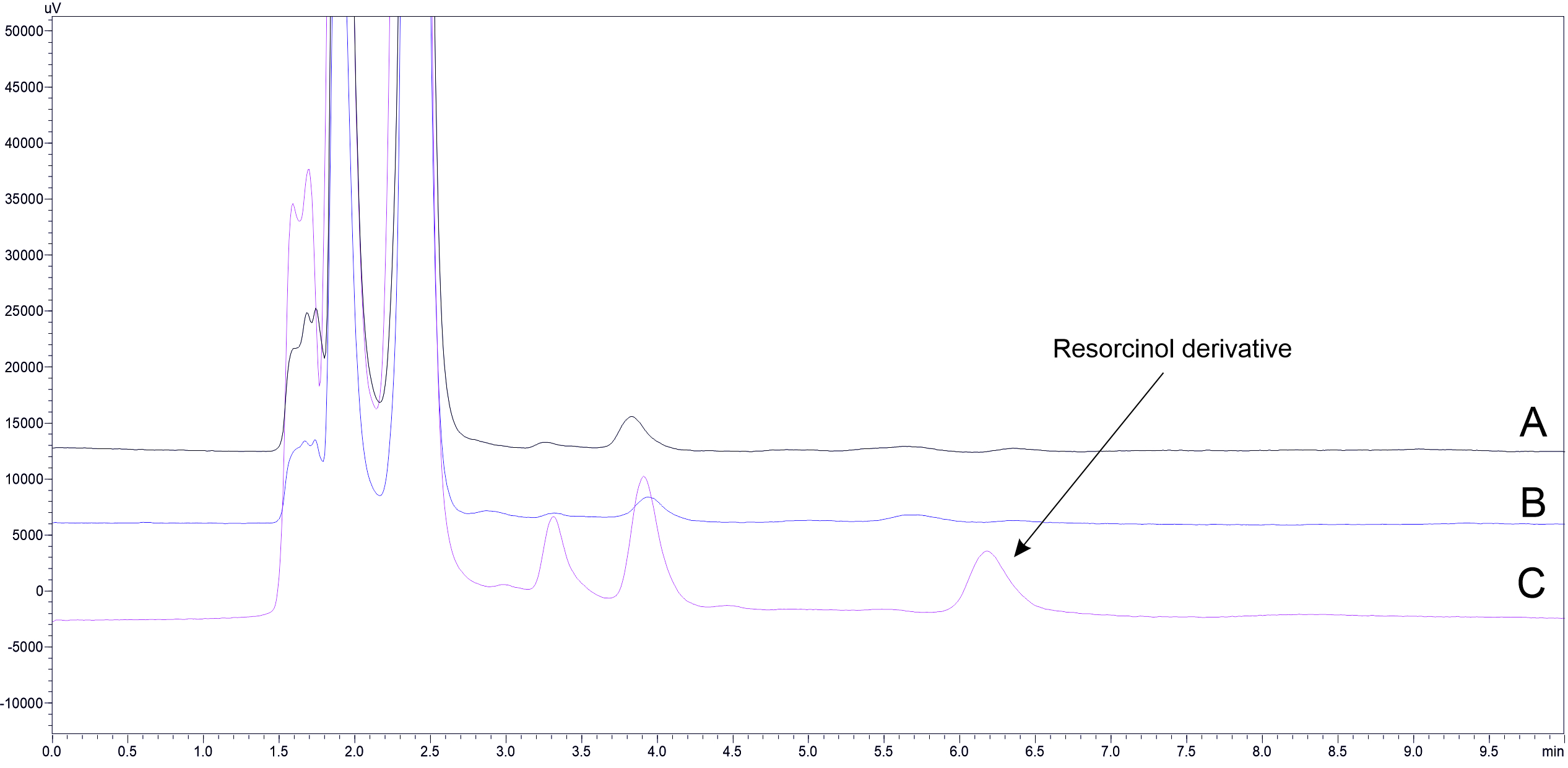


**Figure S4.** HPLC-FLD chromatograms of the analysis of resorcinol-free hair dye A) unspiked sample (Brand A), B) unspiked sample (Brand B) and C) sample (Brand A) spiked with 5 ng/mL (250 μg/g) resorcinol.


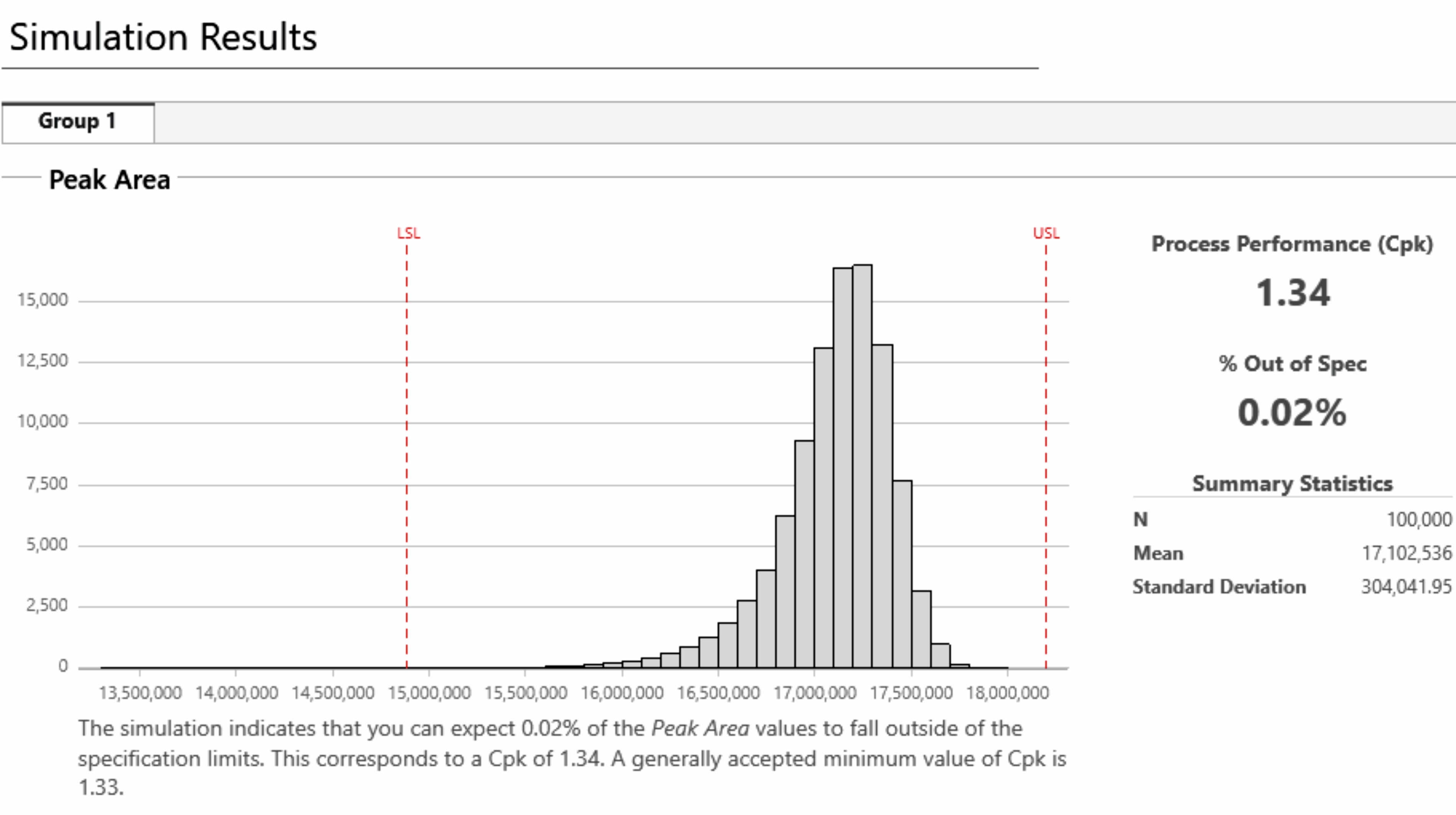


**Figure S5.** Probabilistic distribution of the peak area of azamonardine derivative during Monte-Carlo simulation experiments.
